# Climate‐related changes of soil characteristics affect bacterial community composition and function of high altitude and latitude lakes

**DOI:** 10.1111/gcb.13545

**Published:** 2016-11-25

**Authors:** Carina Rofner, Hannes Peter, Núria Catalán, Fabian Drewes, Ruben Sommaruga, María Teresa Pérez

**Affiliations:** ^1^ Lake and Glacier Ecology Research GroupInstitute of EcologyUniversity of InnsbruckTechnikerstraße 25InnsbruckAustria; ^2^ Limnology, Department of Ecology and GeneticsUniversity of UppsalaUppsalaSweden; ^3^ Catalan Institute for Water Research (ICRA)Emili Grahit 101Girona17003Spain; ^4^ Present address: Stream Biofilm and Ecosystem Research LaboratoryÉcole Polytechnique Fédérale de Lausanne (EPFL)LausanneSwitzerland; ^5^ Present address: ARGE LimnologieAngewandte Gewässerökologie GesmbHInnsbruckAustria

**Keywords:** allochthonous organic carbon, bacterial production, dissolved organic matter, diversity, heterotrophic, phosphorus limitation, terrestrial vegetation, treeline

## Abstract

Lakes at high altitude and latitude are typically unproductive ecosystems where external factors outweigh the relative importance of in‐lake processes, making them ideal sentinels of climate change. Climate change is inducing upward vegetation shifts at high altitude and latitude regions that translate into changes in the pools of soil organic matter. Upon mobilization, this allochthonous organic matter may rapidly alter the composition and function of lake bacterial communities. Here, we experimentally simulate this potential climate‐change effect by exposing bacterioplankton of two lakes located above the treeline, one in the Alps and one in the subarctic region, to soil organic matter from below and above the treeline. Changes in bacterial community composition, diversity and function were followed for 72 h. In the subarctic lake, soil organic matter from below the treeline reduced bulk and taxon‐specific phosphorus uptake, indicating that bacterial phosphorus limitation was alleviated compared to organic matter from above the treeline. These effects were less pronounced in the alpine lake, suggesting that soil properties (phosphorus and dissolved organic carbon availability) and water temperature further shaped the magnitude of response. The rapid bacterial succession observed in both lakes indicates that certain taxa directly benefited from soil sources. Accordingly, the substrate uptake profiles of initially rare bacteria (copiotrophs) indicated that they are one of the main actors cycling soil‐derived carbon and phosphorus. Our work suggests that climate‐induced changes in soil characteristics affect bacterioplankton community structure and function, and in turn, the cycling of carbon and phosphorus in high altitude and latitude aquatic ecosystems.

## Introduction

Climate change alters ecosystem properties and thereby threatens biodiversity and ecosystem functioning. Lake ecosystems integrate changes in the surrounding landscape and atmosphere, acting as sentinels of climate change (Sommaruga‐Wögrath *et al*., 1997; Carpenter *et al*., 2007; Pham *et al*., 2008; Williamson *et al*., 2008; Adrian *et al*., 2009). Several physical properties of lakes tend to respond directly to climatic forcing, such as water temperature and the duration of ice cover (Adrian *et al*., 2009; O'Reilly *et al*., 2015). Chemical lake characteristics often integrate catchment processes such as rock weathering and terrestrial primary productivity. Ecosystem properties, such as primary and bacterial secondary productivity, diversity of taxa, and taxa turnover integrate both physical and chemical alterations over time (Wagner & Adrian, 2009).

Lakes at high altitude and latitude are particularly vulnerable to climate change (Woodward *et al*., 2010). These remote ecosystems are typically small, oligotrophic, and ice‐covered for several months, amplifying the direct and indirect effects of climate change (Grimm *et al*., 2013). Compared to an expected global mean surface temperature increase of 1.0–3.7 °C until the end of the 21st century, surface temperatures in alpine and subarctic regions are expected to increase by ~3–6 °C (Beniston, 2005) and 2.2–8.3 °C (Collins *et al*., 2013), respectively. Moreover, snowpack duration is projected to decrease (Räisänen & Eklund, 2011; Steger *et al*., 2012), whereas the frequency and magnitude of extreme precipitation or drought events will increase (Fischer & Knutti, 2015). Finally, climate change induces range expansions of many plant and animal taxa at their altitudinal and latitudinal extremes (Grabherr *et al*., 1994; Lenoir *et al*., 2008; Harsch *et al*., 2009). Such shifts in local plant cover and plant community composition (Sturm *et al*., 2001; Parmesan & Yohe, 2003; Hinzman *et al*., 2005; Lenoir *et al*., 2008; Pearson *et al*., 2013), and their consequences on soil organic matter content and composition (Reuss *et al*., 2010; Classen *et al*., 2015), are well documented. However, observational and experimental knowledge on the effects of the treeline shift and the concomitant changes in soil chemical characteristics on lake ecosystems at high altitude and latitude regions is missing.

Heterotrophic bacteria play a central role in the cycling of terrestrial organic carbon and nutrients in inland waters (Jones, 1992; Tranvik, 1992; Bergström & Jansson, 2000). The quantity of carbon incorporated to bacterial biomass or released through respiration is determined by the bioavailability of organic matter and the ability of microorganisms to degrade this heterogeneous mixture (Del Giorgio & Cole, 1998; Guillemette & Del Giorgio, 2011). Extensive work on the quantity and quality of terrestrial organic matter in lake ecosystems revealed that the degree of afforestation or catchment composition controls the characteristics of the soil organic matter sources and thus, the rates of carbon processing (e.g., Bastidas Navarro *et al*. 2014; Berggren *et al*. 2010; Forsström *et al*. 2013; Judd *et al*. 2006; Kritzberg *et al*. 2004; Roiha *et al*. 2011). In turn, bacterial communities have been shown to respond quickly to variation in substrate availability and concentration, often favouring opportunistic bacteria such as members of *Betaproteobacteria* (Burkert *et al*., 2003; Šimek *et al*., 2005; Hornák *et al*., 2006; Posch *et al*., 2007) or *Bacteroidetes* (Cottrell & Kirchman, 2000; Battin *et al*., 2001; Eiler *et al*., 2003; Zeder *et al*., 2009). Besides the critical role of heterotrophic bacteria in processing soil‐derived organic matter in inland waters, knowledge on covarying factors, such as the release from nutrient limitation during pulses of soil run off in high altitude and latitude lakes remains limited (Pérez & Sommaruga, 2006).

Here, our aim was to assess how bacterial communities in high altitude and latitude lakes respond to alterations in soil organic matter composition as a response to an upward shift in vegetation. The experiments were conducted as pulse additions of soil‐derived organic matter from the local catchment (i.e., above treeline) compared to additions of soil extracts from a nearby catchment below the treeline. We hypothesized that lake bacteria will respond rapidly (within hours) to organic matter additions and that different taxa will prevail depending on the origin of the soil source. We expected lake communities to be readily adapted to inputs from local catchments, thus initially abundant taxa being able to utilize these soil sources. In contrast, addition of soil extracts from below the treeline may stimulate the growth of initially rare bacterial taxa, potentially causing shifts in community structure. Moreover, we hypothesized that soil extracts will relieve phosphorus limitation and may lead to a reduction in bacterial diversity with fast‐growing taxa dominating bacterial community composition.

## Materials and methods

In summer 2014, an *in situ* experiment was done in one alpine and in one subarctic oligotrophic lake, located above and north of the current treeline, respectively. In both experiments, the *in situ* microbial community was incubated for 72 h and amended with two different soil extracts, namely, one from the lake own catchment above the treeline and one from a nearby site located below the treeline.

### Study sites

The subarctic, oligotrophic lake Saanajärvi (SAA; Fig. S1a) is located at 679 m a.s.l. in north‐western Finland (69°05′N 20°87′E). This medium‐sized lake (70 ha) has a steep shore, a maximum depth of 24 m, and is ice‐covered for up to 9 months per year. The catchment (461 ha) mainly comprises steep mountain slopes and consists of bare rocks (limestone and calcareous dolomite) and a thin soil layer. Plant diversity is rich and dominated by dwarf shrubs, lichens and subarctic flowering plants. More information on SAA and the Kilpisjävi region can be found in Sorvari (2001). The alpine lake Gossenköllesee (GKS; Fig. S1b) is located at 2417 m a.s.l. in the Austrian Alps (47°13′N 11°01′E). Gossenköllesee is small (1.7 ha) with a maximum depth of 9.9 m and usually ice‐covered for up to 7 months per year. The catchment (30 ha) comprises crystalline bedrock, scarce vegetation and a poor soil layer.

### Soil extract preparation

For each experiment, soil extracts from the local catchment (local soil) and from the catchment of a lake located below the treeline (hereafter, foreign soil) were prepared according to Kalbitz *et al*. (2003). Foreign soil extracts were obtained from the lake catchments of Bajit lvgujärvi, Norway (69°21′N 20°19′E) and Piburgersee, Austria (47°11′N 10°53′E) (see Table S1 for key physicochemical parameters). Soils were sampled from six sites in each catchment. The uppermost soil layer, stones, roots and large organic debris were removed, and in total, 4 kg of soil was soaked in 4 L Milli‐Q water for 18–19 h at 4 °C. Then, the water–soil mixture was passed through a cotton cloth and centrifuged at 2 500–10 000 g for 20–30 min. The supernatant was sequentially filtered through 3‐μm (Polycarbonate, Millipore, Vienna, Austria), 1.2‐μm (GF/C, Whatman, GE Healthcare, Vienna, Austria), 0.7‐μm (GF/F, Whatman, GE Healthcare), 0.5‐μm (GF‐5, Marchery Nagel, Düren, Germany), 0.45‐μm (Cellulose ester, Millipore) and 0.2‐μm (Cellulose acetate, Sartorius, Vienna, Austria) filters to obtain a bacteria‐free soil extract (cross‐checked by DAPI‐staining and epifluorescence microscopy). Nutrient and DOC concentrations in the soil extracts were analysed as described later on.

### Experimental set‐up

Water was sampled from 1‐m depth from a boat anchored above the deepest point of the lakes and filtered through 1.0‐μm (Polycap 75 filter column, Whatman, GE Healthcare) using a peristaltic pump (Model Vampire, Bürkle, Bad Bellingen, Germany) to obtain the bacterioplankton fraction. The filtered water (10 L) was collected in HCl‐washed and Milli‐Q‐rinsed polycarbonate bottles for SAA, and in polyethylene bottles for GKS. Triplicates were prepared for both soil extract additions and the control (no soil extract addition). The bottles containing the filtrates were allowed to acclimatize for 24–40 h in the lakes prior to the soil extract additions. Between 213 and 438 mL of soil extracts were added to 10 L of filtered lake water to increase the DOC concentration by a factor of 3. The bottles were placed in wooden racks or steel mesh and incubated at the northern lake shore in SAA and at the eastern lake shore in GKS.

The bottles (*n* = 9) were sampled at the beginning of the experiment (*t* = 0), after 6, 24 and 72 h, thus reducing the end volume to ca. 3 L. The water samples were collected into HCl‐ and Milli‐Q‐rinsed polyethylene bottles and subsequently split to measure bulk uptake rates (^33^P‐orthophosphate, ^33^P‐ATP and ^3^H‐leucine), taxon‐specific uptake using the same substrates (MAR‐CARD‐FISH; only in SAA) and basic physicochemical parameters. Furthermore, 500 mL were filtered on 0.2‐μm filter (GPWP, Millipore) and stored at −80 °C for molecular analyses, and 10 mL subsamples were fixed with 3.7% formaldehyde (final concentration) and stored at 4 °C to assess bacterial abundance by flow cytometry.

### DOC and nutrient analysis

For SAA, water chemical analyses were run by the METLA Institute in Rovaniemi, Finland. Briefly, samples for total dissolved phosphorus (TDP) and inorganic phosphate (Pi‐P) analyses were filtered through 0.4‐μm (Track‐Etched polycarbonate, Nuclepore) and analysed on a Lachat QuikChem 8 000 automated ion analyzer. Dissolved organic carbon (DOC) and total dissolved nitrogen (TDN) concentrations of the soil extracts and SAA lake water samples were determined on a Shimadzu TOC‐L_CSH/CSN,_ while samples taken during the experiment in SAA and the GKS experiment were filtered through two precombusted (450 °C for 4 h) filters (GF/F, Whatman, GE Healthcare), adjusted to pH 2 (HCl) and analysed on a Shimadzu TOC‐V_CPH_ series. Total dissolved phosphorus concentrations for the GKS experiment were estimated by the molybdenum blue method (Vogler, 1966). Dissolved organic matter (DOM) was characterized by means of absorbance and fluorescence spectroscopy, and details can be found in the supplementary information.

### DNA extraction and 16S rRNA gene amplicon sequencing

DNA was extracted using a commercial kit (PowerWater DNA Isolation Kit, MOBIO, Carlsbad, CA, USA), and samples were shipped to LGC Genomics (Germany) for library preparation and 300‐bp paired‐end sequencing (Miseq V3, Illumina, San Diego, CA, USA). There, the V2–V3 region of the bacterial 16S rRNA gene was PCR‐amplified using barcoded (7 bp) primers (341F‐785R) (Klindworth *et al*., 2013). The PCR products were quality‐screened and equimolar‐mixed. A total of 20.1 million reads were obtained from a single MiSeq run and de‐multiplexed using bcl2fastq 1.8.4 software (Illumina). Reads were sorted allowing for no mismatch in barcode sequences, and primer and barcodes were clipped from the sequences. Forward and reverse reads were merged, and the sequence quality was controlled using mothur (Kozich *et al*., 2013). Sequences with more than two ambiguous bases, more than eight homopolymers and shorter than 400 bp were discarded. The sequences were aligned against a reference database (SILVA Release 123), and sequences with <4 different bases were merged using mothur's pre.cluster algorithm. Chimeric sequences were identified using mothur's UCHIME implementation. Finally, singletons and sequences not classified as bacteria by mothur's Naïve Bayesian classifier were also removed from the data set. In total, 1 864 746 sequences remained after quality screening. Sequences were clustered into operational taxonomic units (OTUs) based on the neighbour joining method of mothur's cluster algorithm using a 97% similarity cut‐off. As samples obtained in our experiments had different numbers of sequences, the data set was rarefied to 10 000 sequences to allow for comparability of diversity estimates between samples. Samples with <10 000 sequences and samples which appeared to be outliers (i.e. two samples derived from GKS resembled more communities found in SAA than all other samples from GKS) were removed. As two of three replicates of the foreign soil treatment in GKS at time point 72 h were compromised, we removed this data point from downstream analyses. Sequences have been deposited at the Sequence Read Archive under accession number: SRP077041.

### Incubations for microautoradiography (MAR)

For microautoradiography, 10‐mL samples were incubated at *in situ* temperatures with one of the following substrates (Perkin Elmer): ^33^P‐Pi (specific activity 155.8 Ci mg^−1^; final concentration 40 pM), ^33^P‐ATP as model compound for dissolved organic phosphorus (specific activity 3000 Ci mmol^−1^; final concentration 100 pM) and ^3^H‐leucine as indicator for bacterial production (specific activity 53 Ci mmol^−1^; final concentration 20 nm). Samples for ^3^H‐leucine uptake were incubated for 2 h, while the uptake of ^33^P‐Pi and ^33^P‐ATP was stopped after 3 h by adding formaldehyde (2% final conc.). The fixed samples were kept at 4 °C overnight and then filtered onto white 0.22‐μm polycarbonate filters (GTTP, Millipore). Filters were rinsed with 10 mL sterile filtered double‐distilled water and stored at −20 °C until further processing. The most dominant bacterial taxa in the lakes were targeted by catalysed‐reporter‐deposition‐fluorescence‐*in*‐*situ*‐hybridization (CARD‐FISH; *Alphaproteobacteria*,* Betaproteobacteria*,* Bacteroidetes*, and the AcI lineage of *Actinobacteria*). We also targeted important members within the *Betaproteobacteria* such as the R‐BT cluster (lineage of genus *Limnohabitans*). Information on filter preparation and processing for MAR and CARD‐FISH can be found in the supplementary information.

### Substrate bulk uptake rates

Incubations for ^33^P‐Pi, ^33^P‐ATP and ^3^H‐leucine bulk uptake rates were performed as described for MAR. Additionally, one formaldehyde‐killed blank was fixed 15 min before adding the radioactive substrate. Incubations lasted for 45 min in the case of ^33^P‐Pi and ^33^P‐ATP, and for 1 h for ^3^H‐leucine and were terminated by adding formaldehyde. Samples were filtered onto white 0.22‐μm polycarbonate filters (Poretics). The ^33^P incubations were filtered within 10–60 min to minimize isotope leakage, and the samples for leucine uptake were extracted with trichloroacetic acid (5%) for 5 min and rinsed with the same solution. Filters were placed in scintillation vials with 5 mL of scintillation cocktail (Ready‐safe, Beckman Coulter, Brea, CA, USA). The radioactivity of the filters was assessed after 16 h on a scintillation counter (LS 6 000IC, Beckman Coulter).

### Bacterial abundance

Bacterial abundance was assessed by flow cytometry according to Del Giorgio *et al*. (1996). Briefly, formaldehyde‐fixed cells were stained with 2.5 μm SYTO13 which binds to DNA and RNA. The cells were identified in plots of fluorescence at 520 nm vs. side scatter of a 488‐nm laser on a MoFlo Astrios (Beckman Coulter). Bacterial abundance was calculated from the ratio of cells to 1‐μm fluorescent reference beads (Sigma Aldrich, Vienna, Austria), which were counted under an epifluorescence microscope and adjusted for dilution due to the addition of fixative and dye.

### Statistics

Multivariate statistical analyses such as analysis of similarity (ANOSIM) were performed using r (R Development Core Team, 2015) package ‘vegan’ (Oksanen *et al*., 2011), and phylogenetic diversity was estimated using package ‘picante’ (Kembel *et al*., 2010). A one‐way analysis of variance (anova) or a several sample repeated measure test were run on Past (Hammer *et al*., 2001) to detect whether soil extract additions caused significant differences in the proportions of cells taking up the substrates or in the bulk substrate uptake rates. Sample means were compared between treatments and time. When significant differences (*P *<* *0.05) were found, a post hoc test (Tukey) was applied. Normal distribution of data was visually checked with histograms, normal probability plots and Shapiro–Wilk test. When not normally distributed, data was log‐transformed.

## Results

### Water chemistry and environmental parameters

**Figure 1:**
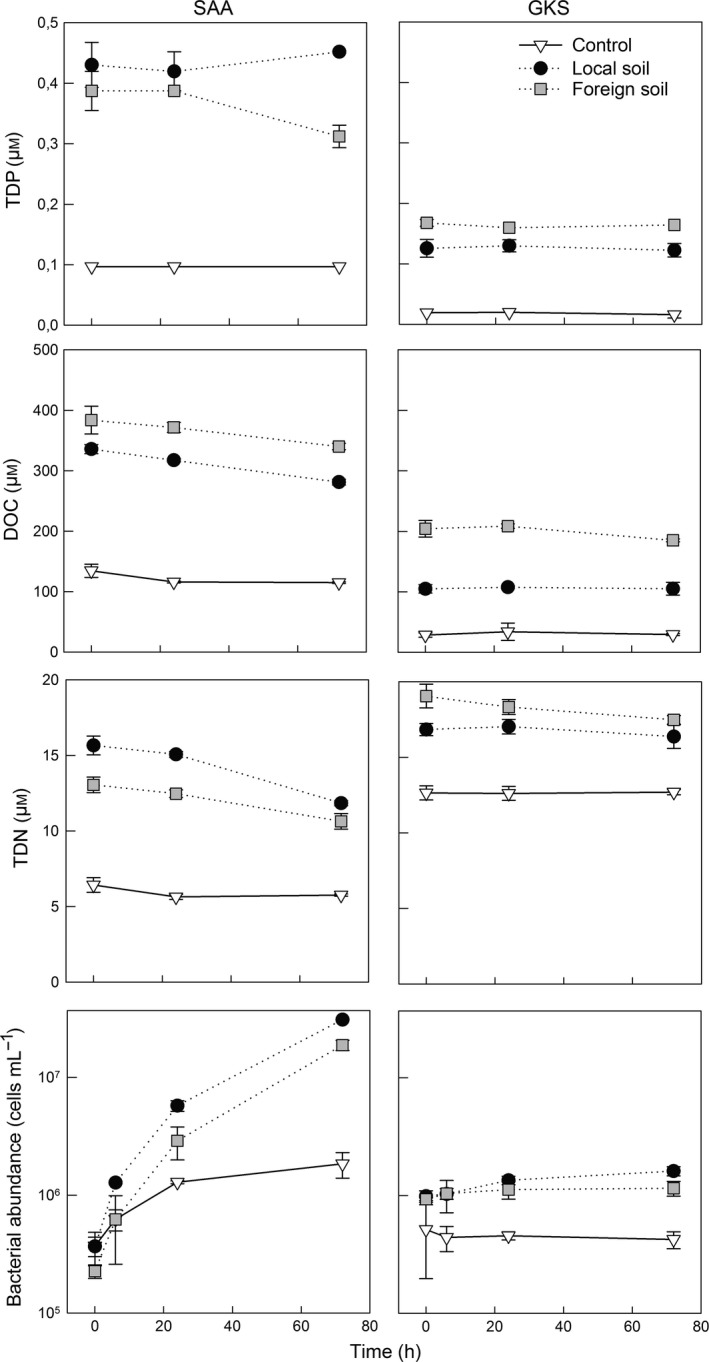
Temporal changes in chemical parameters and bacterial abundance. Different symbols indicate the control and the two treatments in lakes SAA and GKS for which total dissolved phosphorus (TDP), dissolved organic carbon (DOC), total dissolved nitrogen (TDN) and bacterial abundance were determined. Each data point represents the average of triplicate incubations. Error bars represent ± 1 SD, and in some cases are smaller than the symbol. Note that bacterial abundance is given on a logarithmic scale.

Soil extract additions increased the dissolved organic carbon (DOC) concentration by a factor 2.5–3 in SAA, and by a factor 3 or 7 in GKS (Fig. 1). In the SAA experiment, DOC concentrations gradually decreased in all incubations, whereas in GKS, concentrations remained constant or slightly decreased (Fig. 1; foreign soil treatment). The soil extract additions increased the initial total dissolved phosphorus (TDP) concentrations (0.10 μm) to 0.41 ± 0.03 μm in SAA; and from 0.02 μm to 0.15 ± 0.03 μm in GKS (Fig. 1). In SAA, TDP concentrations increased slightly to 0.45 μm in the local soil treatment and decreased to 0.31 μm in the foreign soil treatment by the end of the experiment. In GKS, TDP concentrations remained rather constant during the experiment in all treatments (Fig. 1). Inorganic phosphate (Pi‐P) was only determined in the SAA experiment and decreased from 0.12 to 0.06 μm and 0.18 to 0.10 μm in the local and foreign soil treatments, respectively. Total dissolved nitrogen (TDN) concentrations were more than double in the soil treatments than in the control in SAA, and about one‐third higher in GKS. In all soil treatments, TDN concentrations decreased steadily until the end of the experiment (Fig. 1). The soil extracts changed the relative contribution of chromophoric DOM and of the terrestrial to microbial‐derived organic matter (Table S2). Lake water temperature increased from 13 to 18 °C during the experiment in SAA (Table S1), whereas water temperature remained low (i.e., between 10 and 11 °C) throughout the experiment in GKS.

### Bacterial community structure and succession

Bacterial abundance increased in the course of the experiment in SAA (Fig. 1), but this increase was more pronounced in soil treatments (85‐fold) compared with the control (5‐fold). Bacterial abundance in GKS remained rather constant in the control, but increased by a factor 1.6 in the local and foreign soil treatments (Fig. 1).

**Figure 2:**
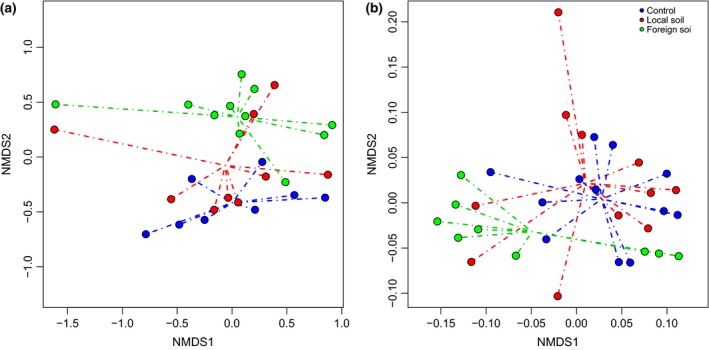
Nonmetric multidimensional scaling plot based on Bray–Curtis similarity in lakes (a) SAA and (b) GKS. Filled circles show communities in the control, the local soil and the foreign soil treatments. Lines connect the single communities to the centroids of each treatment.

Bacterial community composition based on 16S rRNA gene amplicon sequencing differed significantly between treatments and times in GKS (ANOSIM, *R* = 0.23, *P *<* *0.01), but not in SAA (ANOSIM, *R* = −0.02, *P *=* *0.60). However, nonmetric multidimensional scaling plots (Fig. 2) showed that in both lakes, bacterial community composition overlapped in the controls and the local soil treatments, whereas the foreign soil treatments showed more separation.

**Figure 3:**
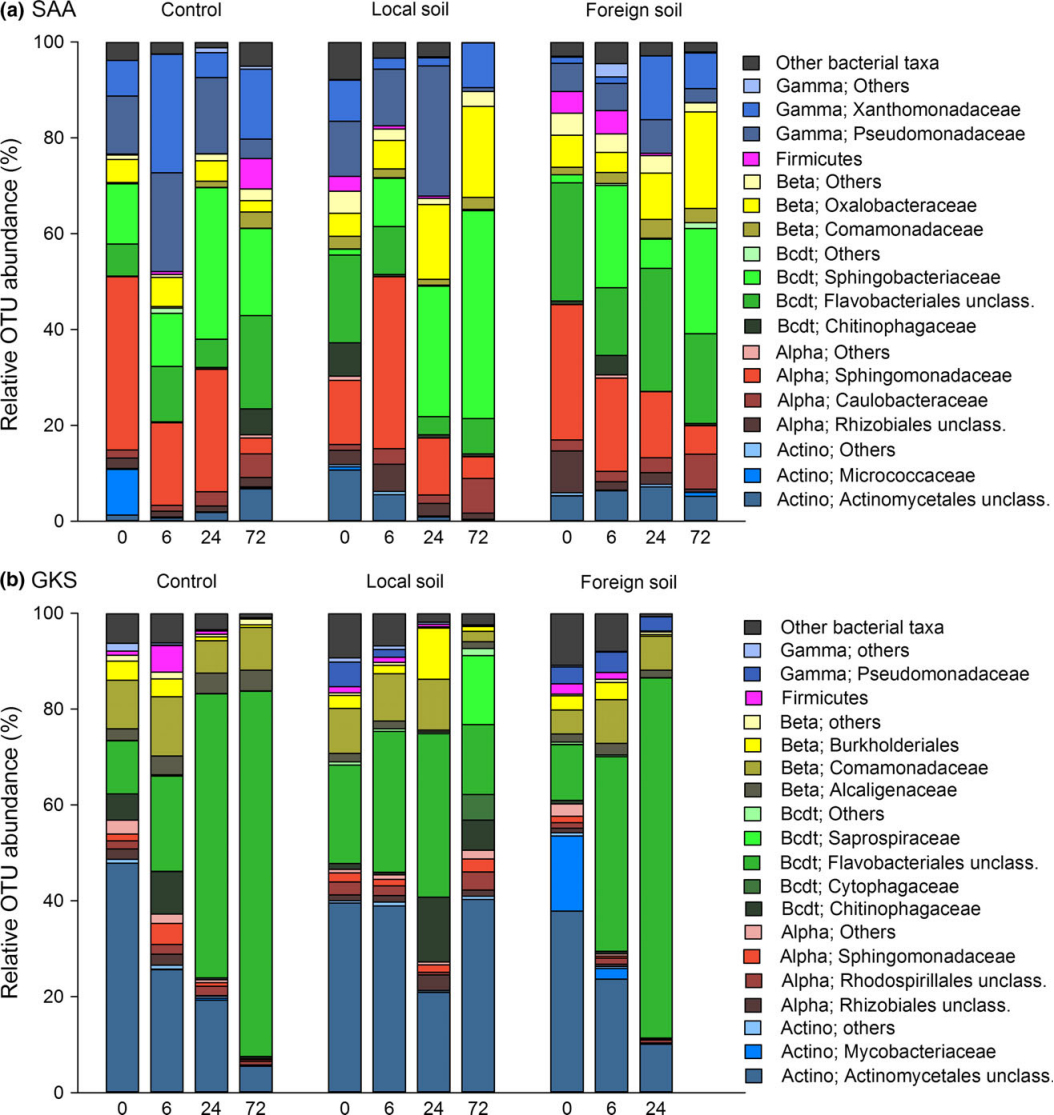
Community composition at the family level in (a) SAA and (b) GKS. Relative OTU abundance from left to right, in the control, the local and the foreign soil treatments determined at every sampling point (0, 6, 24, 72 h). Bacterial taxa that contributed most to the total number of sequences per sample were *Gammaproteobacteria* (Gamma), Firmicutes, *Betaproteobacteria* (Beta), *Bacteroidetes* (Bcdt), *Alphaproteobacteria* (Alpha) and *Actinobacteria* (Actino). Unclassified bacteria and other typical freshwater taxa that accounted for <3% of total sequence number were categorized as ‘Others’.

Bacterial community composition in the two lakes was very different at the beginning of the experiments (Fig. 3). In SAA, bacterial sequences were dominated by different classes of *Proteobacteria* and in GKS, mainly by *Actinobacteria*. During the course of the experiments, a bacterial succession was observed in the controls and the soil treatments of both lakes (Fig. 3, Figs S2 and S4), where initially rare OTUs belonging to *Bacteroidetes* increased in relative abundance. In SAA, members of *Sphingobacteriaceae* and *Flavobacteriales* dominated (Fig. 3a), whereas in GKS mainly *Flavobacteriales* were responsible for the enrichment (Fig. 3b). At the end of the experiments (72 h), *Bacteroidetes* accounted for 30–60% of the bacterial assemblage in the soil treatments, but taxa related to *Betaproteobacteria* (*Oxalobacteraceae*) and *Actinobacteria* (*Actinomycetales*) also gained in relative abundance (Fig. 3, Figs S2 and S4).

In total, 5 070 OTUs were detected throughout both experiments. In GKS, 297 ± 108 OTUs were detected at the beginning of the experiment, whereas 162 ± 69 OTUs were found in SAA. In all treatments and controls, diversity measured as the number of OTUs, evenness (Shannon H’) and phylogenetic diversity (Faith's pd) gradually decreased during the incubations (Fig. S3). However, when comparing final vs. initial OTU abundances, initially rare bacterial taxa dominated community structure in the soil treatments, whereas in the control, initial dominant taxa prevailed throughout the incubations (Fig. S4).

### Bulk community phosphate, ATP and leucine uptake rates

**Figure 4:**
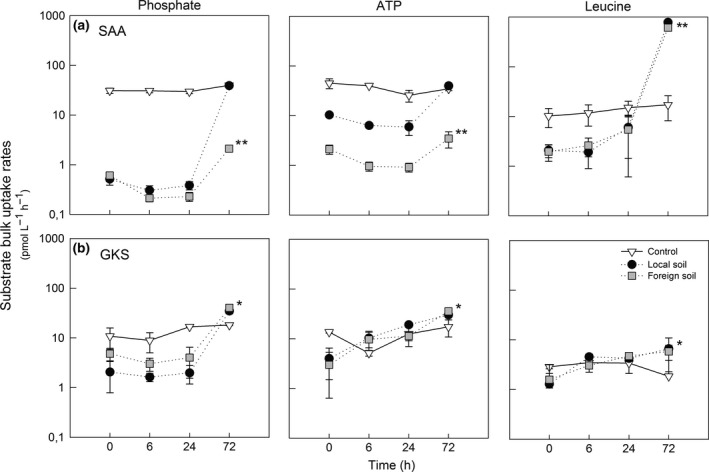
Phosphate, ATP and leucine bulk uptake rates assessed during the experiments. Different symbols indicate the control and the two treatments in lakes (a) SAA and (b) GKS. Data points represent the mean of triplicate incubations, and error bars represent ± 1 SD which are in some cases smaller than the symbol. Asterisks indicate significant difference (Tukey, *P <* *0.01, **P <* *0.001) in substrate bulk uptake rates between the control and the soil treatments at the end of the experiments. Note that the *y*‐axis is in logarithmic scale.

Phosphate, ATP and leucine bulk uptake rates decreased initially in the soil treatments, but increased at the end of the incubations (Fig. 4). In SAA, both soil extract additions initially reduced the uptake of radiolabelled leucine to ~2 pmol L^−1^ h^−1^ (Fig. 4a). However, towards the end of the experiment, these rates increased significantly to 783 and 610 pmol L^−1^ h^−1^ in the local and foreign soil treatments (Tukey, *P *<* *0.001), respectively. During the first 24 h, phosphate uptake rates were two magnitudes lower in the soil treatments (0.38 ± 0.16 pmol L^−1^ h^−1^) when compared to the control (30 ± 0.81 pmol L^−1^ h^−1^), whereas ATP bulk uptake rates ranged between 0.92 and 10.4 pmol L^−1^ h^−1^. At the end of the experiment, Pi and ATP uptake rates in the local soil treatment reached levels similar to the control (Fig. 4a), but these rates remained significantly lower in the foreign soil treatment (Pi: 2.12 pmol L^−1^ h^−1^, ATP: 3.48 pmol L^−1^ h^−1^; Tukey, *P *<* *0.001).

In GKS, leucine uptake rates remained low throughout the experiment in all incubations (<7 pmol L^−1^ h^−1^), but increased significantly in both soil treatments after 72 h (Fig. 4b; Tukey, *P *<* *0.01). Phosphate uptake rates were significantly lower in the local (1.89 ± 0.23 pmol L^−1^ h^−1^) and foreign soil treatments (3.95 ± 0.91 pmol L^−1^ h^−1^) when compared to the control (12.16 ± 4.09 pmol L^−1^ h^−1^) during the first 24 h (Tukey, *P *<* *0.05). At the end, Pi uptake rates in both soil treatments exceeded the control rates, reaching 37.55 ± 3.96 pmol L^−1^ h^−1^. By contrast, ATP bulk uptake rates in the soil treatments exceeded the control rates already after 6 h and were double as high as in the control after 72 h (foreign soil: 35.54 pmol L^−1^ h^−1^; local soil: 30.12 pmol L^−1^ h^−1^).

### Taxon‐specific uptake of phosphate, ATP and leucine in Saanajärvi

**Figure 5:**
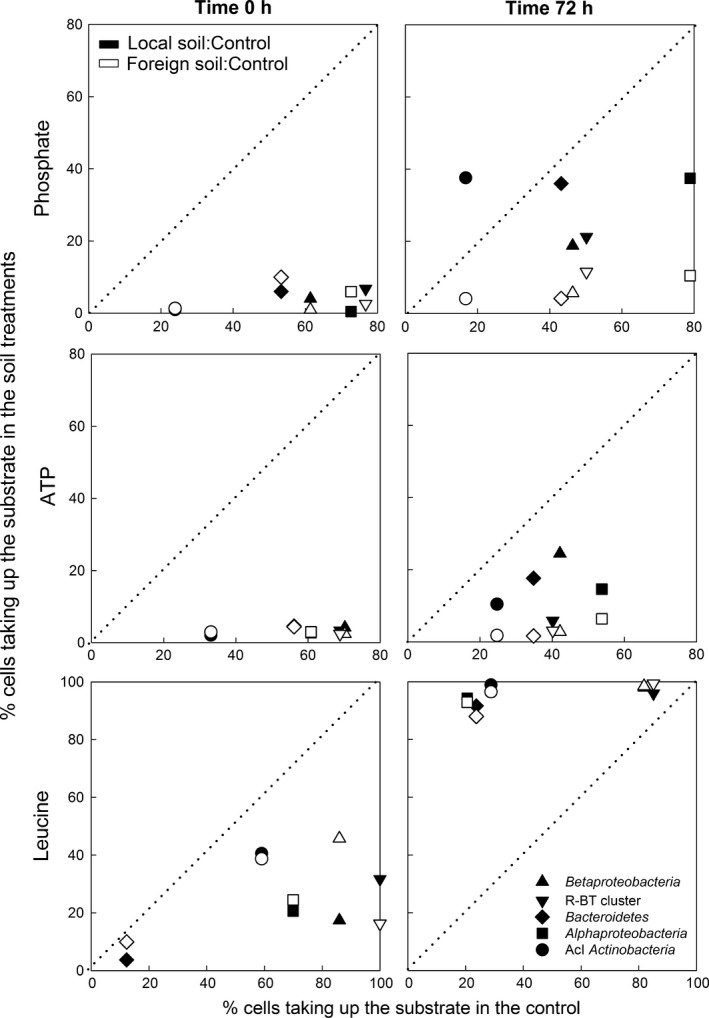
Taxon‐specific uptake of phosphate, ATP and leucine in the soil treatments vs. in the control. Results are given as the percentage of *Betaproteobacteria*, its R‐BT cluster, *Bacteroidetes*,* Alphaproteobacteria*, and AcI *Actinobacteria* taking up the substrates in the local and foreign soil treatment plotted against the control at the beginning (0 h) and the end of the experiment (72 h) in SAA.

In the control and throughout the experiment, the most common bacterial taxa examined using MAR‐CARD‐FISH (*Betaproteobacteria*, its R‐BT cluster, *Bacteroidetes*,* Alphaproteobacteria*) contributed substantially to Pi and ATP uptake (range: 35–88% of hybridized cells), whereas AcI *Actinobacteria* only contributed 14–33% to the uptake of these substrates (Fig. 5, Fig. S5). A large percentage of bacteria from all taxa examined (range: 59–100% of hybridized cells) took up leucine at the beginning of the experiment in the control, except for *Bacteroidetes*. Generally, the proportions of cells taking up a substrate declined throughout the experiment in the control (Fig. 5, Fig. S5).

Soil extract additions caused a significant decrease in the proportions of cells taking up Pi, ATP and leucine (Fig. 5; anova,* P *<* *0.05). These percentages dropped below 10% for Pi and ATP incorporation, whereas the percentage of cells taking up leucine comprised 4–41%. During the experiment, these proportions increased (Fig. S5), but the magnitude of increase depended on soil origin. For instance, cells positive for P uptake increased among most bacterial taxa in the local soil treatment reaching 30.2 ± 9.4% and 14.6 ± 7.1% of cells positive in Pi and ATP uptake, respectively. However, the percentage of Pi‐ and ATP‐labelled cells in the foreign soil treatment remained low (Fig. 5). By contrast, all bacterial taxa examined were significantly overrepresented (data points above the 1 : 1 line) in leucine uptake in both soil treatments when compared with the control at the end of the experiment (Fig. 5; anova,* P *<* *0.01).

**Figure 6:**
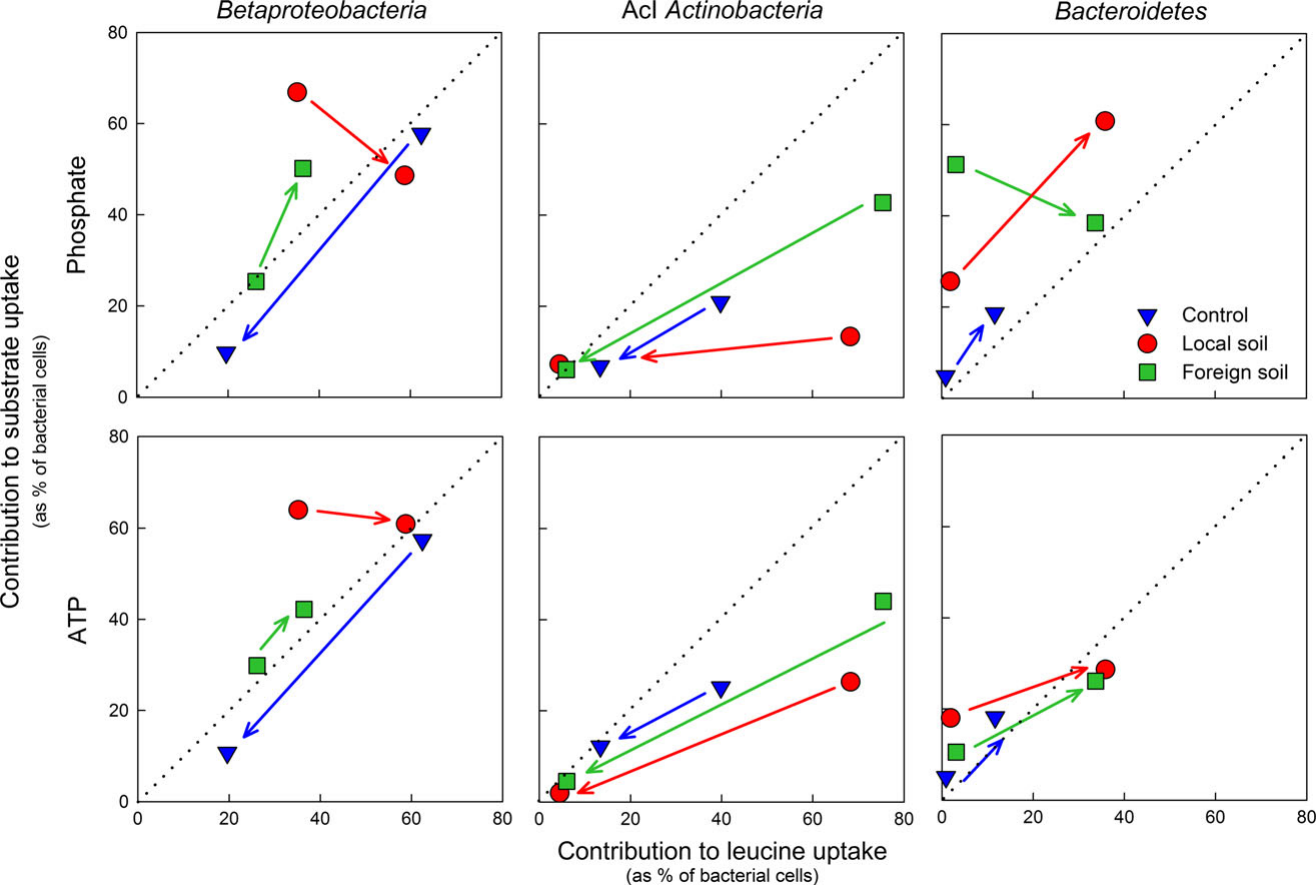
Relative contribution of the dominant bacterial taxa to phosphate and ATP uptake plotted against leucine uptake. Different symbols indicate the control and the two treatments in the SAA experiment. Shifts in contribution to substrate uptake from the beginning of the experiment (0 h) towards the end (72 h) are indicated by arrows. Values are mean of triplicate incubations and the dashed line indicates a 1 : 1 relationship.

In the control, the contribution of a specific bacterial taxon to the uptake of Pi and ATP was generally proportional to its contribution to leucine incorporation (Fig. 6), only AcI *Actinobacteria* were underrepresented in P uptake. The contribution to substrate uptake declined during the experiment in the control for all taxa except *Bacteroidetes,* which increased their contribution to P and leucine uptake. In contrast to the control, we found that soil extract additions caused considerable shifts in the contribution of major taxa to substrate uptake (Fig. 6). For instance, *Betaproteobacteria* were overrepresented for Pi and ATP compared to leucine uptake in the local soil treatment, whereas they contributed equally to substrate uptake in the foreign soil treatment (Fig. 6). By the end of the experiment, this class dominated the uptake of the three substrates in both soil treatments and contributed to P uptake in relation to leucine incorporation. Similarly, *Bacteroidetes* were overrepresented in Pi and ATP uptake when compared to leucine uptake (2.5 ± 0.9%) in both soil treatments at the beginning of the experiment. Towards the end, this phylum increased its relative contribution to cells taking up leucine (34.8 ± 1.5%) and thus contributed to Pi and ATP uptake (38.5 ± 15.8%) proportionally to leucine incorporation. The contribution of *Bacteroidetes* in the local soil treatment was higher for Pi uptake than for leucine one. Although AcI *Actinobacteria* accounted for the majority of cells taking up leucine at the beginning in the soil treatments (Fig. 6), their relative contribution to the uptake of any substrate declined during the experiment and was negligible by the end of it.

## Discussion

### Soil extract additions cause rapid bacterial successions

Soil extract additions strongly affected the structure and abundance of the bacterioplankton community of lakes located above the treeline (Figs 1 and 2, Fig. S3). Shifts in lake bacterial community structure and cell abundance have been previously linked to the source of organic matter entering a lake (Crump *et al*., 2003; Roiha *et al*., 2011; Forsström *et al*., 2013), and our results indicate that changes in soil organic matter quality induce quick shifts in community composition (Figs 2 and 3, Fig. S2).

In the high‐altitude lake GKS, the initial bacterial community was dominated by the members of *Actinobacteria* and *Betaproteobacteria* which agrees with previous reports from mountain lakes in the central Alps (Warnecke *et al*., 2005; Salcher *et al*., 2010; Pérez & Sommaruga, 2011). The contribution of major freshwater phyla to community structure in the high‐latitude lake was rather evenly distributed and represented by classes of *Proteobacteria, Actinobacteria* and *Bacteroidetes*. Upon soil extract additions, bacterial community composition followed successional patterns, with rare taxa rapidly dominating over the initial community, and persistent taxa gaining importance towards the end (Fig. 3, Fig. S2). These patterns were consistent in both the local and the foreign soil treatments, but the increase of initially rare bacteria was more rapid in the foreign soil treatments at both locations. This was probably mediated by a different composition of the soil sources (e.g., low‐ vs. high‐molecular‐weight compounds) (Moran & Hodson, 1990; Berggren *et al*., 2010; Logue *et al*., 2016), which favoured community members having the required metabolic capacities (Judd *et al*., 2006; Logue *et al*., 2016). The foreign soil treatments showed a higher aromatic character, most likely linked to higher lignin‐derived compound concentrations from the forest vegetation, whereas in the local soil treatments, this property was less pronounced (Table S2). The stronger effects on bacterial structure observed in treatments with foreign than local soils were likely due to larger differences in DOM quality characteristics. However, in both soil treatments, the specific and rapid community change, concomitantly with the decrease in diversity (Fig. S3), indicated that soil extract additions selected for copiotrophic bacteria, rare under oligotrophic conditions, but preponderant during periods of high allochthonous loadings. This agrees with previous observations from field (Crump *et al*., 2003; Roiha *et al*., 2011) and laboratory experiments (Logue *et al*., 2016) showing that rapid shifts in community composition take place upon soil organic matter input. The loss and prevalence of the taxa determined in our study may be explained by their different functional adaptability, including differences in resource affinities (Cottrell & Kirchman, 2000; Salcher *et al*., 2011; Heinrich *et al*., 2013), physiological characteristics (Hahn & Pöckl, 2005; Šimek *et al*., 2006) and genetic composition (Bauer *et al*., 2006; Gómez‐Pereira *et al*., 2012; Teeling *et al*., 2012; Tveit *et al*., 2013). Further, changes in lake bacterial community composition have been attributed not only to the differential response of individual bacterial taxa to DOM inputs, but also to the introduction of soil bacteria with allochthonous sources or to incubation effects (Crump *et al*., 2003; Logue *et al*., 2016). In our experiments, however, the addition of soil‐borne bacteria with the soil extracts was not confirmed when using staining techniques and microscopy. Nevertheless, potential bottle effects, which can favour the growth of copiotrophic bacteria (Christian & Capone, 2002), cannot be discarded. In any case, it is evident that bacteria interact in a complex way, partly depending on competition for or facilitation of resource utilization (Fuhrman *et al*., 2015). It can be assumed that the effects of soil source on bacterial community composition and succession were direct and mediated through a combination of ecological lifestyles (copiotrophs vs. oligotrophs) and taxon‐specific substrate preferences (specialists vs. generalists) (Cottrell & Kirchman, 2000; Eiler *et al*., 2003; Salcher *et al*., 2013; Pérez *et al*., 2015), as well as through species interactions.

### Effects of soil extract additions on bacterial P limitation and leucine uptake

The degree to which allochthonous sources support lake ecosystem productivity depends on the availability of limiting nutrients and the bioavailability of the carbon source (Del Giorgio & Cole, 1998; Stets & Cotner, 2008; Berggren *et al*., 2009). Thus, changes in vegetation cover of lake catchments could have severe consequences for the productivity of oligotrophic lakes, if P and DOC availability increases (Jansson *et al*., 2006).

Soil organic matter is often dominated by high‐molecular‐weight (HMW) compounds and thus, an initial step of extracellular enzymatic activity is required to break down these compounds into low‐molecular‐weight (LMW) compounds prior to bacterial uptake (Arnosti, 2003; Cunha *et al*., 2010). In our experiments, soil organic matter additions led to the initial reduction of Pi, ATP and leucine uptake at the community (Fig. 4) and the single‐cell level (Fig. 5). These model compounds are readily accessible for bacteria and hence reflect the uptake of inorganic and organic P, as well as of labile DOC compounds. The initial low substrate uptake rates we observed (Fig. 4) could result from a dilution of the radiolabelled substrates with nonlabelled‐compounds, suggesting that LMW compounds were added with the soil extracts (De Haan & De Boer, 1978; Sweet & Perdue, 1982; Pérez & Sommaruga, 2007; Berggren *et al*., 2010). This trend reversed as the experiment progressed, and substrate uptake rates often reached levels similar to the control or exceeded the control's rates. Nevertheless, the degree of response clearly differed between locations (SAA vs. GKS) and soil treatments (local vs. foreign soil treatment in SAA). The patterns observed in substrate uptake rates were most likely the result of differences in initial bacterioplankton community composition (Logue *et al*., 2016), DOC lability (Kalbitz *et al*., 2003; Berggren *et al*., 2009) and nutrient (or rather P) availability (Moran & Hodson, 1990; Olsen *et al*., 2002). For instance, total dissolved phosphorus concentrations were three times lower in the local and in the foreign soil treatments in GKS than in SAA (Table S1). Furthermore, the higher leucine uptake rates concomitantly with increasing bacterial abundances observed in SAA may not only reflect the compositional differences of the soil amendments, but also the stimulatory effect of the increase in water temperature during the experiment (Table S1), which did not occur in GKS. For example, a 5 °C increase in water temperature in a mountain stream stimulates leucine uptake rates by four times (Tibbles, 1996).

The origin of the soil extract and its effect on bacterial functioning was also reflected by changes in the substrate utilization profiles of individual bacterial taxa in SAA (Figs 5 and 6) and was in agreement with results obtained in GKS previously (Pérez & Sommaruga, 2006, 2007). The substrate uptake patterns suggested that the dominant bacterial taxa contribute differently to the processing of soil‐derived resources in the treatments. For example, considering that the contribution to substrate uptake of some initially abundant taxa decreased (e.g., AcI *Actinobacteria*) suggests that their importance during soil run‐off events in general was low. By contrast, other taxa (e.g., *Betaproteobacteria*) were fundamental for C and P cycling irrespectively of the origin of the soil organic matter (Fig. 6). However, the high representation of rare taxa (e.g., *Bacteroidetes*) in Pi and ATP uptake when compared to leucine incorporation suggests that they accumulated excess P at the beginning of the experiments, probably to sustain fast growth. This agrees with the growth‐rate hypothesis which postulates that fast‐growing organisms have high P requirements (Elser *et al*. 2000) due to the increase of P‐rich cell structures required for growth (e.g., ribosomes; Franklin *et al*. 2011). These results are particularly relevant because the contribution of different taxa to the uptake of C and P substrates greatly affects the energy and nutrient supply to aquatic food webs (Šimek *et al*., 2005; Salcher *et al*., 2007). Our findings agree with a recent study showing that in grazer‐free incubations, and particularly during periods of high organic matter loadings, rare taxa dominated bacterial community composition (Neuenschwander *et al*., 2015). The authors suggested that the low abundance of these taxa at natural settings is controlled by bacterivorous grazing, implying that their biomass contributes substantially to the C channelled towards higher trophic levels. Our results suggest that copiotrophic bacteria do not only channel a substantial fraction of C, but also of P during soil run‐off events.

Given the pivotal role of inland waters in the global carbon cycle and the potentially large number of lakes affected by climate change (Raymond *et al*., 2013), understanding the effects of catchment alterations on the diversity and function of heterotrophic bacterial assemblages in high altitude and latitude lakes is crucial (Judd *et al*., 2006; Forsström *et al*., 2013; Solomon *et al*., 2015). Diverse assemblages of heterotrophic bacteria control the conversion of organic matter into biomass and regulate organic matter mineralization, determining carbon emissions to the atmosphere (Bass *et al*., 2010). Our experiments deal with the initial responses of lake bacterioplankton communities to soil organic matter amendments from above and below the treeline (Fig. S6), a scenario which gains realism given the increasing evidence for range expansion of many plant taxa at their altitudinal and latitudinal extremes (Sturm *et al*., 2001; Parmesan & Yohe, 2003; Hinzman *et al*., 2005; Lenoir *et al*., 2008; Pearson *et al*., 2013). Rare but fast‐growing taxa played a fundamental role in this initial response, but complex interactions formed by initially abundant and persistent bacteria may influence the long‐term effects. Although, the resilience of the communities to changes in soil‐derived inputs and to other climate‐change‐related factors need to be addressed in future experimental work, our results hint to potential major effects on the C and P cycling in lakes affected by the treeline shift (Fig. S6). Factors such as temperature rise or photochemical transformation of allochthonous organic matter (Vähätalo *et al*., 2003) will shape the functional response of bacteria under natural conditions. Further, specific lake characteristics, such as water residence time and ice‐cover duration (Bergström & Jansson, 2000; Adrian *et al*., 2009), and the chemical composition of atmospheric deposition (Kopáček *et al*., 2011) may be important in determining the relevance and magnitude of the response. Finally, the increasing frequency and magnitude of heavy‐precipitation events (Fischer & Knutti, 2015) might foster the input of terrestrial organic matter with strong consequences for lake metabolism and the dominance of heterotrophic processes (Sadro & Melack, 2012; Forsström *et al*., 2013).

## Supporting information


**Figure S1.** The subarctic lake Saanajärvi (SAA) in Finland and the alpine lake Gossenköllesee (GKS) in Austria.
**Figure S2.** Relative abundance of specific bacterial taxa in the control and soil treatments in SAA and GKS.
**Figure S3.** Temporal dynamics of community diversity in SAA and GKS.
**Figure S4.** Initial vs. final sequence abundances in the control and soil treatments in SAA and GKS.
**Figure S5.** The proportions of probe‐specific bacterial taxa taking up phosphate, ATP and leucine in the SAA experiment.
**Figure S6.** Schematic depiction of the effects of climate‐induced change in soil run‐off composition on lake bacterial community composition and functioning.
**Table S1.** Summary of physicochemical and biological parameters determined in lakes SAA and GKS, and during the experiments.
**Table S2.** Optical characteristics of dissolved organic matter measured at the beginning of the experiments.
