# Supplementary material for: Climate‐related changes of soil characteristics affect bacterial community composition and function of high altitude and latitude lakes
Source: Glob Chang Biol. 2016 Nov 25;23(6):2331–44. doi: 10.1111/gcb.13545 (PMC5434934; doi:10.1111/gcb.13545)

**Supplementary Experimental Procedures and Information**

**CAtalyzed-Reporter-Deposition-Fluorescence-*In*-*Situ*-Hybridization (CARD-FISH) and MicroAutoRadiography (MAR) analysis**

Samples for CARD-FISH were processed according to Pernthaler *et al.* (2002) with the modified permeabilization step described by Sekar *et al.* (2003). Briefly, horseradish peroxidase-labelled rRNA probes (ThermoHybaid) were used to target the domain *Bacteria* (EUB I-III (Daims *et al.*, 1999)), *Alphaproteobacteria* (ALF968 (Neef, 1997)), *Betaproteobacteria* (BET42a (Manz *et al.*, 1992)), and its R-BT cluster (R-BT065 (Šimek *et al.*, 2001)), *Bacteroidetes* (CF319a (Manz *et al.*, 1996)), and the AcI lineage of *Actinobacteria* (AcI-852 (Warnecke *et al.*, 2005)). After hybridization (4-6 h at 35°C) and signal amplification (30-45 min at 37°C), one filter half was counterstained with 4’,6-diamidino-2-phenylindole (DAPI) and prepared for microscopy, whereas the other half was further processed for microautoradiography.

Microautoradiography (MAR) was done after transferring cells onto cover slips (Cottrell & Kirchman, 2000, 2003). The cover slips were dipped in 2:1 diluted autoradiography emulsion (Kodak, type NTB) and developed according to the manufacturer’s instructions after 9 h exposure for the ^33^P-substrates and 19 h exposure for the ^3^H-leucine samples. The slides were counterstained with DAPI (1 µg mL^-1^) and examined under an epifluorescence microscope (Axiophot 2, Zeiss, Germany). For each sample, at least 100 CARD-FISH-labelled cells were counted and checked for silver grain accumulation using transmission light. Cells surrounded by at least two silver grains were considered as MAR+ cells. For CARD-FISH, about 400 DAPI-stained cells per sample were counted and checked for probe-specific signals. In total, 192 filter sections were prepared for CARD-FISH and 576 filter sections for MAR-CARD-FISH.

**Dissolved organic matter (DOM) characterization**

Absorbance spectra (200 to 800 nm) for DOM characterization were measured at 1 nm intervals with a Lambda 40 spectrophotometer (Perkin-Elmer, Waltham, USA). The absorption coefficients (aλ) and the slopes (S) of the absorbance spectra at different wavelength ranges were determined according to Stedmon *et al.* (2000). The slope ratio SR, calculated as the ratio of S275-295/S350-400, is related to the dominant DOM molecular weight (Helms *et al.*, 2008). Fluorescence excitation - emission matrices (EEM) were obtained with a spectrofluorometer (SPEX Fluoromax-4, Horiba Jobin Yvon). Excitation wavelengths ranged from 250 nm to 445 nm at intervals of 5 nm, and emission wavelengths from 300 nm to 600 nm at increments of 4 nm. The EEMs were corrected for Raman scattering, inner-filter effects (Kothawala *et al.*, 2013, McKnight *et al.*, 2001) and normalized to Raman units (R.U.) (Cory & McKnight, 2005), using the FDOM correct toolbox (Murphy *et al.*, 2010). We calculated several indices derived from the EEMs, including the freshness or biological index (BIX) according to Parlanti *et al.* (2000), the Fluorescence Index (FI, Jaffé *et al.* (2008)) and the humification index (HIX).

**References**

Cory RM, Mcknight DM (2005) Fluorescence spectroscopy reveals ubiquitous presence of oxidized and reduced quinones in dissolved organic matter. *Environmental Science & Technology,* **39**, 8142–8149.

Cottrell MT, Kirchman DL (2000) Natural assemblages of marine Proteobacteria and members of the *Cytophaga-Flavobacter* cluster consuming low- and high-molecular-weight dissolved organic matter. *Applied and Environmental Microbiology,* **66**, 1692-1697.

Cottrell MT, Kirchman DL (2003) Contribution of major bacterial groups to bacterial biomass production (thymidine and leucine incorporation) in the Delaware estuary. *Limnology and Oceanography,* **48**, 168-178.

Daims H, Bruhl R, Amann R, Schleifer K, Wagner M (1999) The domain-specific probe EUB338 is insufficient for the detection of all Bacteria: development and evaluation of a more comprehensive probe set. *Systematic and Applied Microbiology,* **22**, 434-444.

Helms JR, Stubbins A, Ritchie JD, Minor EC, Kieber DJ, Mopper K (2008) Absorption spectral slopes and slope ratios as indicators of molecular weight, source, and photobleaching of chromophoric dissolved organic matter. *Limnology and Oceanography,* **53**, 955-969.

Jaffé R, Mcknight D, Maie N, Cory R, Mcdowell WH, Campbell JL (2008) Spatial and temporal variations in DOM composition in ecosystems: The importance of long-term monitoring of optical properties. *Journal of Geophysical Research,* **113**.

Kothawala DN, Murphy KR, Stedmon CA, Weyhenmeyer GA, Tranvik LJ (2013) Inner filter correction of dissolved organic matter fluorescence. *Limnology and Oceanography: Methods,* **11**, 616-630.

Manz W, Amann R, Ludwig W, Vancanneyt M, Schleifer K (1996) Application of a suite of 16S rRNA-specific oligonucleotide probes designed to investigate bacteria of the phylum Cytophaga-Flavobacter-Bacteroides in the natural environment. *Microbiology,* **142**, 1097-1106.

Manz W, Amann R, Ludwig W, Wagner M, Schleifer K (1992) Phylogenetic oligodeoxynucleotide probes for the major subclasses of Proteobacteria: problems and solutions. *Systematic and Applied Microbiology,* **15**, 593-600.

Mcknight DM, Boyer EW, Westerhoff PK, Doran PT (2001) Spectrofluorometric characterization of dissolved organic matter for indication of precursor organic material and aromaticity. *Limnology and Oceanography,* **46**, 38-48.

Murphy R, Straebler S, Cooper Z, Fairburn CG (2010) Cognitive behavioral therapy for eating disorders. *Psychiatric Clinics of North America,* **33**, 611-627.

Neef A (1997) Anwendung der in situ Einzelzell-Identifizierung von Bakterien zur Populationsanalyse in komplexen mikrobiellen Biozönosen. PhD thesis. Technical University of Munich.

Parlanti E, Wörz K, Geoffroy L, Lamotte M (2000) Dissolved organic matter fluorescence spectroscopy as a tool to estimate biological activity in a coastal zone submitted to anthropogenic inputs. *Organic Geochemistry,* **31**, 1765-1781.

Pernthaler A, Pernthaler J, Amann R (2002) Fluorescence in situ hybridization and catalyzed reporter deposition for the identification of marine bacteria. *Applied and Environmental Microbiology,* **68**, 3094-3101.

Sekar R, Pernthaler A, Pernthaler J, Warnecke F, Posch T, Amann R (2003) An improved protocol for quantification of freshwater *Actinobacteria* by fluorescence in situ hybridization. *Applied and Environmental Microbiology,* **69**, 2928-2935.

Šimek K, Pernthaler J, Weinbauer MG *et al.* (2001) Changes in bacterial community composition and dynamics and viral mortality rates associated with enhanced flagellate grazing in a mesoeutrophic reservoir. *Applied and Environmental Microbiology,* **67**, 2723-2733.

Stedmon CA, Markager S, Kaas H (2000) Optical properties and signatures of chromophoric dissolved organic matter (CDOM) in Danish coastal waters. *Estuarine Coastal and Shelf Science,* **51**, 267-278.

Warnecke F, Sommaruga R, Sekar R, Hofer JS, Pernthaler J (2005) Abundances, identity, and growth state of Actinobacteria in mountain lakes of different UV transparency. *Applied and Environmental Microbiology,* **71**, 5551-5559.

Figure S1. The subarctic lake Saanajärvi in Finland (a) and the alpine lake Gossenköllesee in Austria (b). Source: Carina Rofner.


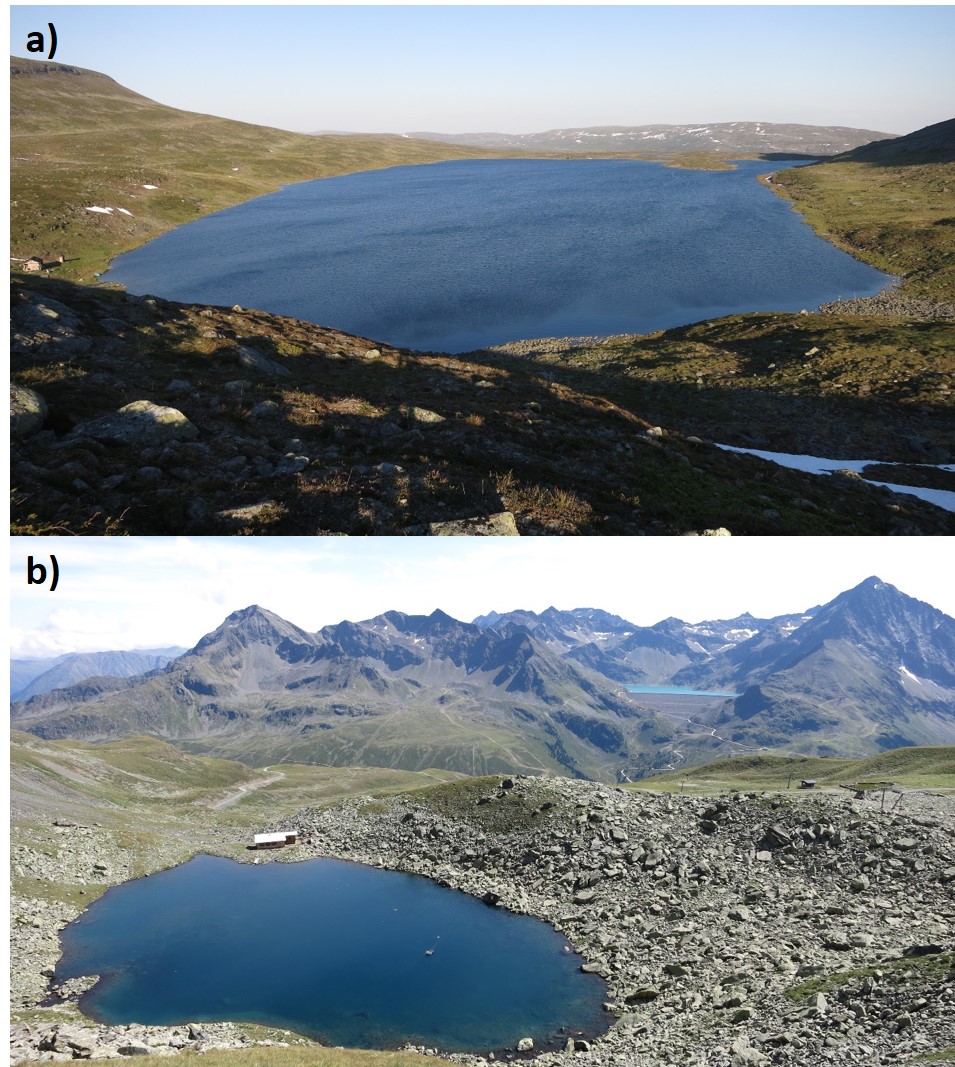


Table S1. Summary of physicochemical and biological parameters of the lakes, the soil extracts, and the enrichment experiments at the beginning (0) and at the end (72 h). SAA: Saanajärvi; GKS: Gossenköllesee; PIB: Piburgersee; T: Temperature; Cond: Specific electrical conductivity at 25°C; DOC: Dissolved organic carbon; TDN: Total dissolved nitrogen; TDP: Total dissolved phosphorus; Pi-P: Inorganic phosphate; BA: Bacterial abundance; Note that TDP and Pi-P in the Saanajärvi experiment were measured by a different method than in the Gossenköllesee experiment.

| Habitat | Date | Sample |  |  | T (°C) | | pH | | Cond (µS cm^-1^) | | DOC (µM) | | TDN (µM) | | TDP (µM) | | Pi-P (µM) | | BA (10^6^ cells ml^-1^) | |
| --- | --- | --- | --- | --- | --- | --- | --- | --- | --- | --- | --- | --- | --- | --- | --- | --- | --- | --- | --- | --- |
|  |  |  |  |  | 0 | 72 | 0 | 72 | 0 | 72 | 0 | 72 | 0 | 72 | 0 | 72 | 0 | 72 | 0 | 72 |
| Catchment SAA | 24.06.14 | Local soil extract, above the treeline |  |  |  |  | 6.57 |  | 17 |  | 6660.56 |  | 431.93 |  | 25.83 |  | 13.72 |  |  |  |
| Catchment Bajit | 26.06.14 | Foreign soil extract, below the treeline |  |  |  |  | 5.50 |  | 29 |  | 11739.24 |  | 332.69 |  | 30.67 |  | 17.76 |  |  |  |
| Lake SAA | 07.07.14 | 1 m |  |  | 7.5 |  | 7.13 |  | 38 |  | 105.74 |  | 8.07 |  | 0.10 |  | <0.06 |  |  |  |
| Experiment | 12-15.07.14 | Control |  |  | 13.0 | 18.0 | 7.08 | 7.13 | 38 | 38 | 134.60 | 115.03 | 6.42 | 5.76 | 0.10 | 0.10 | <0.06 | <0.06 | 0.370 | 1.85 |
|  |  | Local soil extract |  |  | 13.0 | 18.0 | 7.02 | 7.09 | 39 | 39 | 335.75 | 281.16 | 15.65 | 11.83 | 0.43 | 0.45 | 0.12 | 0.06 | 0.367 | 30.9 |
|  |  | Foreign soil extract |  |  | 13.0 | 18.0 | 7.03 | 7.06 | 39 | 39 | 383.73 | 340.08 | 13.05 | 10.63 | 0.39 | 0.31 | 0.18 | 0.10 | 0.226 | 18.8 |
| Catchment GKS | 28.07.14 | Local soil extract, above the treeline |  |  |  |  | 5.00 |  |  |  | 2073.37 |  | 139.14 |  | 9.10 |  |  |  |  |  |
| Catchment PIB | 21.05.14 | Foreign soil extract, below the treeline |  |  |  |  | 6.00 |  |  |  | 11792.52 |  | 426.93 |  | 12.91 |  |  |  |  |  |
| Lake GKS | 21.07.14 | 1 m |  |  |  |  | 7.27 |  | 22 |  | 29.93 |  | 13.31 |  | 0.02 |  |  |  |  |  |
| Lake GKS | 17.08.14 | 1 m |  |  | 10.0 |  | 7.10 |  | 21 |  | 27.81 |  | 12.28 |  | 0.02 |  |  |  |  |  |
| Experiment | 19-22.08.14 | Control |  |  | 10.6 | 10.5 | 7.00 | 6.68 | 22 | 22 | 28.54 | 29.34 | 12.64 | 12.69 | 0.02 | 0.02 |  |  | 0.515 | 0.423 |
|  |  | Local soil extract |  |  | 10.6 | 10.5 | 6.94 | 7.73 | 22 | 22 | 104.96 | 105.13 | 16.83 | 16.37 | 0.13 | 0.12 |  |  | 0.987 | 1.611 |
|  |  | Foreign soil extract |  |  | 10.6 | 10.5 | 6.90 | 6.64 | 23 | 24 | 204.29 | 185.16 | 19.05 | 17.47 | 0.17 | 0.16 |  |  | 0.929 | 1.154 |

Table S2. Optical characteristics of dissolved organic matter measured at the beginning of the experiments. Absorbance coefficients measured at 254 nm and 400 nm reflected the chromophoric character of the soil amendments and was highest in foreign soil treatments. Results of spectrofluorometric analyses such as the Fluorescence Index (FI), Biological Index (BIX) and Humification Index (HIX) show that soil amendments changed the relative contribution of humic substances of terrestrial origin and of microbial-derived organic matter.

|  |  | A_254_ | A_400_ | FI | BIX | HIX |
| --- | --- | --- | --- | --- | --- | --- |
| SAA | Control | 3.80 ± 0.12 | 1.04 ± 0.18 | 1.43 ± 0.04 | 0.65 ± 0.06 | 2.42 ± 0.08 |
|  | Local soil treatment | 7.61 ± 0.99 | 2.12 ± 0.32 | 1.50 ± 0.05 | 0.51 ± 0.06 | 2.43 ± 0.58 |
|  | Foreign soil treatment | 12.99 ± 0.78 | 3.55 ± 0.57 | 1.36 ± 0.04 | 0.52 ± 0.04 | 3.06 ± 1.19 |
| GKS | Control | 1.05 ± 0.12 | 0.37 ± 0.22 | 1.58 ± 0.07 | 0.90 ± 0.05 | 0.27 ± 0.05 |
|  | Local soil treatment | 1.87 ± 0.21 | 0.68 ± 0.16 | 1.53 ± 0.04 | 0.65 ± 0.03 | 0.94 ± 0.10 |
|  | Foreign soil treatment | 5.13 ± 0.27 | 1.40 ± 0.23 | 1.40 ± 0.03 | 0.52 ± 0.04 | 2.40 ± 0.24 |

Figure S2. Relative abundance (as percentage of total prokaryotic cell abundance or DAPI cells) of *Betaproteobacteria*, its R-BT cluster, *Bacteroidetes*, *Alphaproteobacteria*, AcI *Actinobacteria* and other bacteria (using probe EUBI-II-III) in the control and soil treatments in lakes **(a)** SAA and **(b)** GKS determined by CARD-FISH. In GKS, the community structure at t=0 was assessed for the control and at t=72 for every treatment.


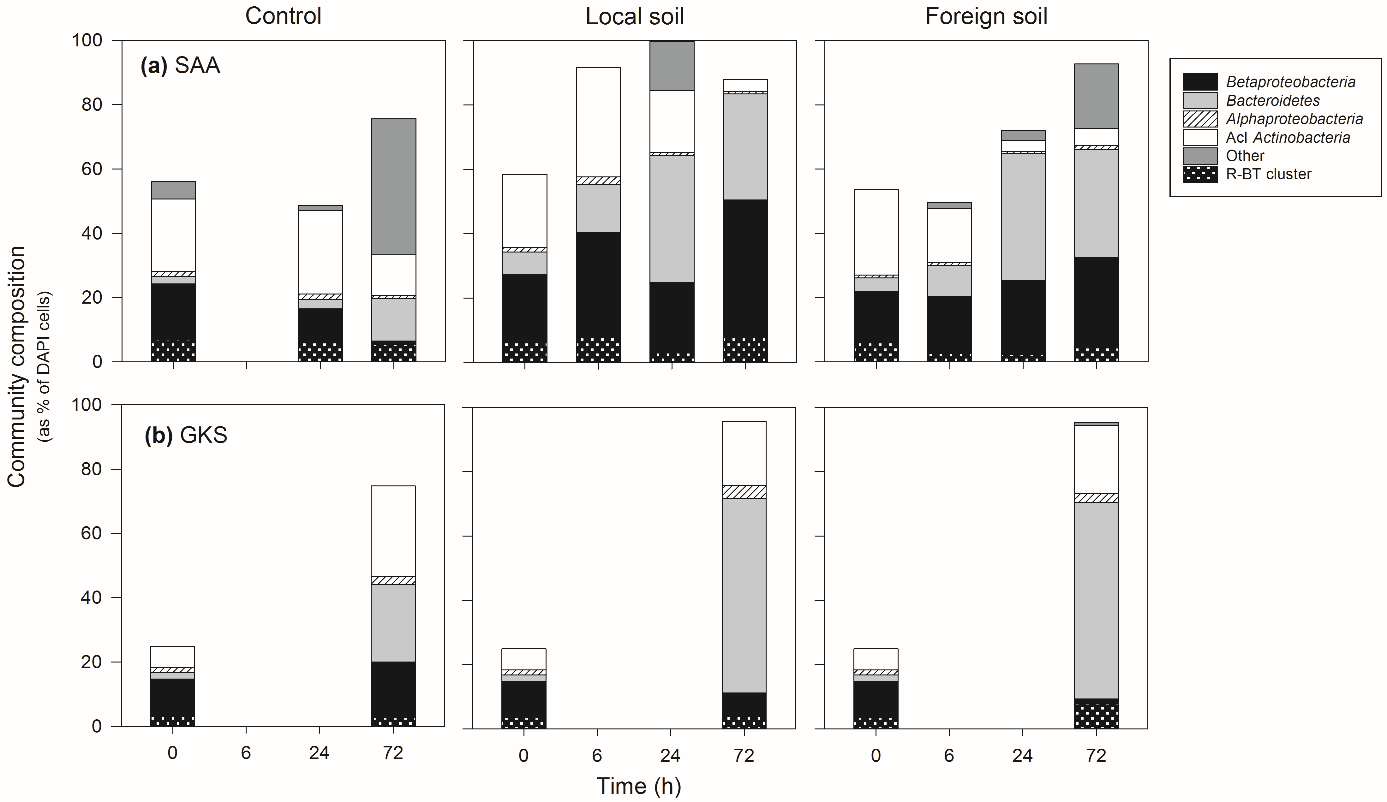


Figure S3. Temporal dynamics of community diversity measured as the OTU richness, Shannon H’ and Faith’s phylogenetic diversity in the control and treatments in lakes Saanajärvi and Gossenköllesee.

Figure S4. Scatterplots of initial (0 h) vs. final (72 h) sequence abundances in the control (a, b), local (c, d) and foreign soil (e) treatments in lakes Saanajärvi and Gossenköllesee. Colours represent phylogenetic class affiliation. Note that samples from Gossenköllesee (foreign soil treatment, t=72 h) could not be visualized since two out of three replicates had to be removed from the dataset.

Figure S5. The proportions of probe-specific bacterial taxa taking up phosphate, ATP and leucine (as percentage of hybridized cells) in the SAA experiment. Black bars indicate the control, open bars the local soil treatment and grey bars the foreign soil treatment. MAR-CARD-FISH was examined at t=0, 6, 24, 72 h and bars are arranged in this order. Each data point represents the average of three incubations. Error bars represent ± 1 SD.


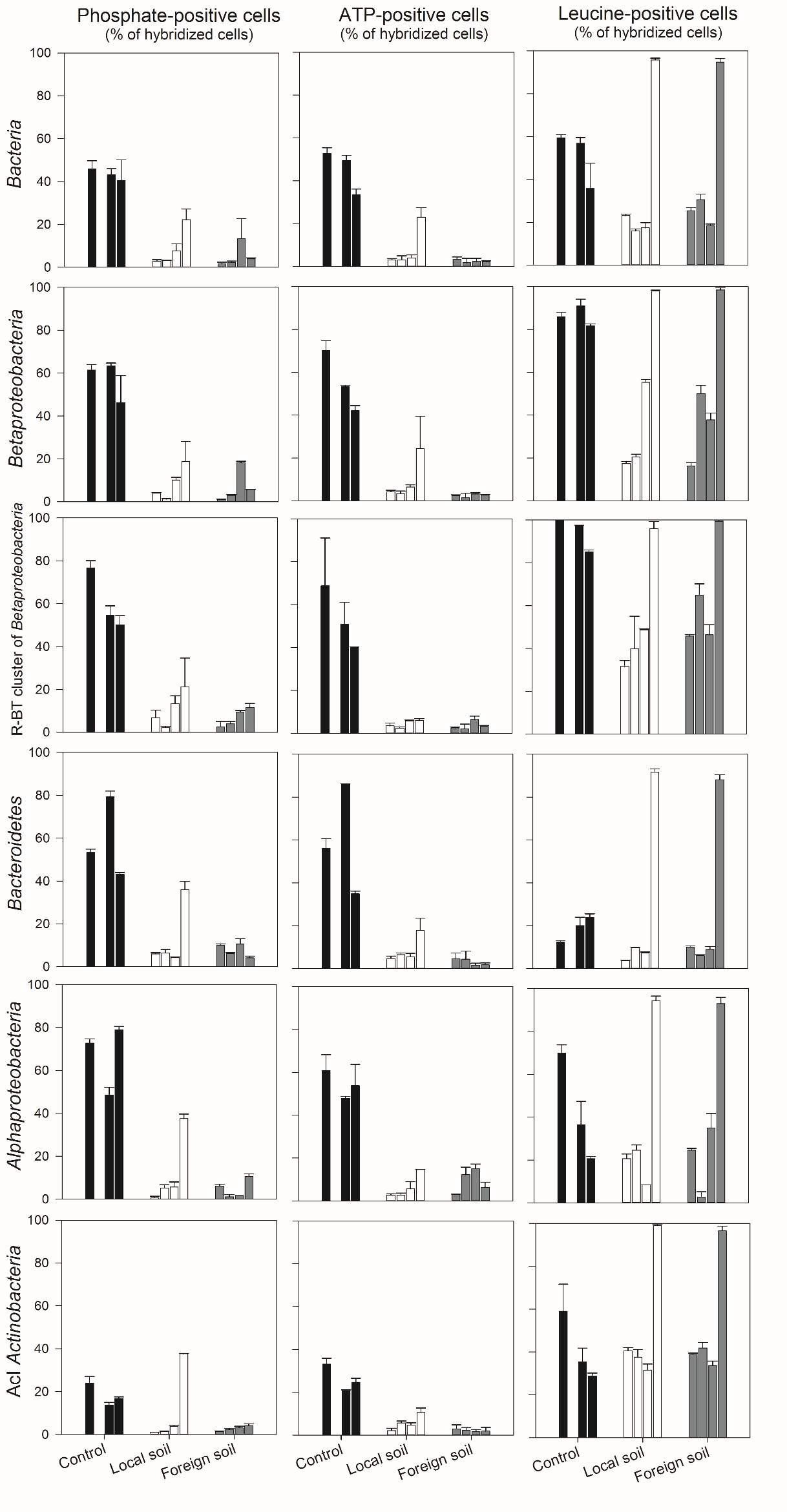


Figure S6. Schematic depiction of the effects of climate-induced change in soil runoff composition on lake bacterial community composition and functioning. The upper panel represents the current condition of an oligotrophic lake above the treeline. Bacterial diversity is high, but bacterial production is limited by dissolved organic phosphorus (DOP) and dissolved inorganic phosphorus (DIP) (dashed lines). The lower panel shows a scenario where climate change affects lake catchment characteristics and thus, soil runoff composition. Soil-derived resources alleviate P-limitation and rapidly growing taxa dominate community composition by consuming labile DOC.


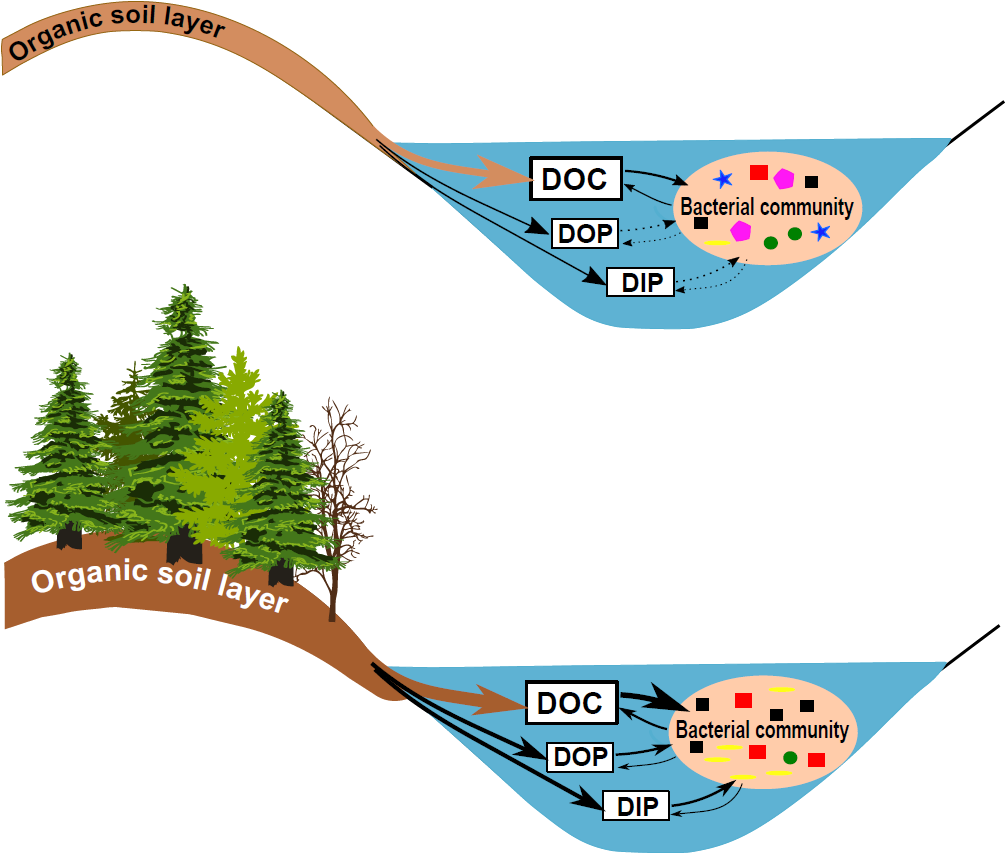

Supplement: Supplementary file 1 — Figure S1. The subarctic lake Saanajärvi (SAA) in Finland and the alpine lake Gossenköllesee (GKS) in Austria. Figure S2. Relative abundance of specific bacterial taxa in the control and soil treatments in SAA and GKS. Figure S3. Temporal dynamics of community diversity in SAA and GKS. Figure S4. Initial vs. final sequence abundances in the control and soil treatments in SAA and GKS. Figure S5. The proportions of probe‐specific bacterial taxa taking up phosphate, ATP and leucine in the SAA experiment. Figure S6. Schematic depiction of the effects of climate‐induced change in soil run‐off composition on lake bacterial community composition and functioning. Table S1. Summary of physicochemical and biological parameters determined in lakes SAA and GKS, and during the experiments. Table S2. Optical characteristics of dissolved organic matter measured at the beginning of the experiments. [file GCB-23-2331-s001.docx]
